# Changes in Young Swimmers’ In-Water Force, Performance, Kinematics, and Anthropometrics over a Full Competitive Season

**DOI:** 10.5114/jhk/183065

**Published:** 2024-05-17

**Authors:** Catarina Costa Santos, Daniel Almeida Marinho, Mário Jorge Costa

**Affiliations:** 1 Department of Sport Sciences, University of Beira Interior, Covilhã, Portugal.; 2 Research Center in Sports Sciences, Health Sciences and Human Development (CIDESD), Covilhã, Portugal.; 3 Centre of Research, Education, Innovation, and Intervention in Sport (CIFI2D), Faculty of Sport, University of Porto, Porto, Portugal.

**Keywords:** swimming, longitudinal, training periodization, biomechanics, sensors

## Abstract

The aim of the present study was to analyze changes in young swimmers’ in-water force, performance, kinematics, and anthropometrics during one full competitive season. Twenty-five swimmers (11 girls and 14 boys, 12.04 ± 0.16 years) were assessed over four distinct time points throughout a competitive season. The in-water force of both hands (D, dominant; ND, non-dominant) was retrieved during two bouts of 25 m front crawl allowing the estimation of the symmetry index. The velocity (v25) was calculated from the time to complete the 25 m and considered the performance outcome, while the stroke rate, stroke length, and the stroke index were used as kinematic variables. For anthropometric variables, body mass, stature, arm span and the hand surface area were measured. The in-water force (16–24%) and performance (8%) improved over the competitive season with significant changes in the first macrocycle. The stroke index was the only kinematic variable that changed between M_1_ and M_4_ (12.7%), accompanied by a higher asymmetric motion later in the season. A time effect was found in the stature (p < 0.001, η_p_^2^ = 0.71), the arm span (p < 0.001, η_p_^2^ = 0.79), and the hand surface area (D = p < 0.001, η_p_^2^ = 0.63; ND = p < 0.001, η_p_^2^ = 0.666). Swimming performance showed associations with in-water force, stroke efficiency and anthropometric features in all time points of the season. Thus, the natural anthropometric growth experienced over the season may translate into a more efficient swimming pattern with greater in-water forces that can enhance performance.

## Introduction

Young swimmers’ performance is characterized by a multifactorial and dynamic phenomenon, where anthropometric and biomechanical characteristics (kinematics or hydrodynamics) define the energetic profile and may contribute to a performance enhancement (Morais et al., 2021). For instance, variables within the biomechanical domain seem to contribute approximately 50–60% to performance (Morais et al., 2012). Although research using young swimmers has been largely focused on the biomechanical field, most of the previous studies presented a cross-sectional research design denoting a lesser understanding about the cause-and-effect relationships than longitudinal designs (Costa et al., 2012a). Thus, monitoring long-term changes in swimming, at least in this age cohort, can be a more useful approach to understand how swimmers progress within the season, and how the training process triggers effects in the various domains of performance.

Swimmers typically undergo an annual traditional training periodization with two or three peak forms (i.e., macrocycles). As they go through a growth and biological maturation process, training programs are mainly focused on the acquisition of fundamental motor skills (Lang and Light, 2010; Martindale et al., 2005). Growth spurts usually occur at some point in the competitive season (Abbott et al., 2021) and are expected to induce changes in other performance-related variables. To date, the few available longitudinal studies in young swimmers were mainly directed toward assessing anthropometrics (Fiori et al., 2022b; Lätt et al., 2009a, 2009b), energetics (Zacca et al., 2020), kinematics and efficiency (Fiori et al., 2022a; Lipińska et al., 2011; P Morais et al., 2013) or dry-land strength/power (Batalha et al., 2013). Young swimmers are prone to improve kinematics along with an increase in anthropometric traits (Jaworski et al., 2020; Lätt et al., 2009a, 2009b; Morais et al., 2013). Improvements in energetics and efficiency also contribute to performance enhancement, mostly in middle-distance events (Ferreira et al., 2021; Zacca et al., 2020). While several changes can happen from the beginning until the end of the season, impairment in stroke mechanics can be seen at specific time points (Morais et al., 2013). Thus, performance should be seen as dynamic, and any shift within a season may be dependent on the training program, swimmers' sex, growth or maturational status.

The most accepted deterministic models point out the influence of anthropometrics and kinetics on swimming kinematics (Barbosa et al., 2013). It means that swimming velocity depends on the interaction of propulsive and drag forces being the in-water force influenced by the swimmer’s technique and strength levels. Thus, the in-water force determines the overall stroke mechanics and as a consequence performance, especially in sprint events (Gatta et al., 2016). The number of studies related to long-term changes in the in-water force of young swimmers is low, and the existent approaches just complied dry-land training, tapering, and warm-up effects over short periods of training (Santos et al., 2021). To date, there is a gap in the literature about the follow up of young swimmers in-water forces and their (non) linear fluctuations during a full competitive season. As the ability to apply force could be fundamental to swimmers' displacement through the water, a deeper understanding of how in-water forces change at different training stages over a season is a welcome approach.

The present study aimed to analyze the effects of a full traditional competitive season on the in-water force, performance, kinematics, and anthropometrics of young swimmers. It was hypothesized that performance and kinematics would improve over the competitive season due to the enhancement of in-water forces and the natural anthropometric growth.

## Methods

### Participants

Twenty-five young swimmers (11 girls and 14 boys; 12.04 ± 0.16 years) were recruited from a local swimming squad. Swimmers had more than two years of competitive experience (regional or national events) and trained four to six times per week. At least two of these in-water training session were preceded by a strength and conditioning session with body weight as the load. Swimmers who did not attend all data collection moments or suffered an injury after the beginning or during the competitive season were excluded. All potential benefits and experimental risks were carefully explained, and swimmers’ parents or guardians signed a written informed consent form. All procedures were approved by the Institutional Ethics Committee of the University of Beira Interior (approval code: CE-UBI-Pj-2020-058; approval date: 21 July 2020) and carried out according to the Declaration of Helsinki.

### Design and Procedures

**Figure 1:**
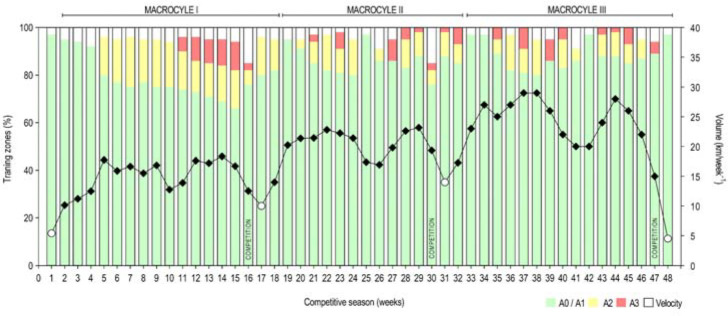
The distribution of training volume (km/week^−1^) and training intensity (%) over the competitive season (three macrocycles). White dots represent the four assessment time points. A0/A1, warm-up, slow pace, and technical drills; A2, medium pace working for anaerobic threshold; A3, intense pace working for VO_2max_; velocity, maximum short distance effort.

A longitudinal follow-up design was selected over one competitive season. Swimmers were evaluated during a traditional training periodization with three peak forms. The evaluation moments (M_i_) were chosen as the beginning of the season (M_1_, September) and after the main competition of the first (M_2_, December), the second (M_3_, April), and the third (M_4_, July) macrocycle. Figure 1 shows the distribution of the training volume (km•week^−1^) and training intensity (%) of the three macrocycles. A single observer measured all anthropometric variables following recommended and standardized protocols (Lohman et al., 1988). All measurements were carried out with swimmers wearing a regular textile swimsuit and a cap. The experimental in-water protocol took place in a 25 m indoor pool with 27.5°C of water temperature. After a standardized warm-up (Morouço et al., 2018), swimmers performed two all-out bouts of 25 m front crawl (maximum intensity) with a full body condition (i.e., upper and lower limbs). Swimmers were instructed to maintain their usual breathing pattern and not to glide after the in-water push-off start.

### Anthropometrics and Biological Maturation

A stadiometer (SECA, 242, Hamburg, Germany) and flexible tape (RossCraft, Canada) were used to measure stature (cm), sitting height (cm), and the arm span (cm). Body mass (kg) was assessed using a digital scale (TANITA, BC-730, Amsterdam, Netherlands) and the body mass index (BMI) was estimated using body mass and the square of body height (kg•m^2^). Hand surface areas (HSAs, cm^2^) were measured by digital photogrammetry (Moreira et al., 2014). Swimmers placed their dominant (D) and non-dominant (ND) hands on a scanning machine and calibration was done as reported elsewhere (Santos et al., 2023). All HSA files were exported and analyzed in specific software (Universal Desktop Ruler, v3.8, AVPSoft, USA). The hand dominance information was obtained by self-report. Before M_1_, the maturity offset (MO) was estimated according to peak height velocity (PHV; Mirwald et al., 2002) and interpreted at the beginning of the season as pre-PHV (< −0.50 years), mid-PHV (−0.50 ≥ MO ≤ 0.50 years) or post-PHV (> 0.50 years).

### Biomechanical and Performance Variables

A reliable (Santos et al., 2022a) and user-friendly (Santos et al., 2022b) differential pressure system (Type A, *f* = 100 Hz, Swimming Technology Research, Richmond, VA, United States) was used to measure in-water kinetics. The two sensors were attached by a cable (15 m of length) to an A/D interface connected to a laptop with the Aquanex software (v.4.1, Model DU2, Swimming Technology Research, Richmond, VA, United States). Before each bout, swimmers kept their hands immersed (10 s) at the waistline to calibrate the system with the hydrostatic pressure values.

Hand resultant force (N) was derived from the product of differential pressures by the HSA of each swimmer. Force-time curves retrieved in all M_i_ were analyzed between the 11^th^ and 24^th^ m section (i.e., 13 m). The mean peak force (F_PEAK_, N) of both hands was defined as the mean of the peak values obtained in all underwater paths of the defined section. The subsequent peak force (SF_PEAK_, N) was defined as the peak value retrieved in two subsequent curves (Santos et al., 2023). Two ground cones were placed to define the section of 13 m and images were recorded using one camera (*f* = 50 Hz, Sony, HDR-CX 240, Japan) in order to avoid considering the stroke cycles made before the 11 m mark. Signal-processing software (AcqKnowledge v.3.7.3, Biopac Systems, Santa Barbara, CA, USA) was used to analyze force data being the signal handled with a 5 Hz cut-off low-pass fourth-order Butterworth filter. The Symmetry Index (SyI, in %) was then estimated using the SF_PEAK_ data of both hands being interpreted as perfect symmetry (SyI = 0%), symmetric motion (0% ≥ SyI ≤ 10%) or asymmetric motion (SyI > 10%). This is a measure that allows to understand force symmetries/asymmetries between both sides of the body in bilateral actions (Robinson et al., 1987).

In-water kinematic and temporal variables were retrieved during the two all-out bouts of 25 m front crawl swimming. Swimming performance (T25, s; ICC: 0.96) was manually assessed (FINIS 3x100, Finis Inc., USA) and the swimming velocity (v25, m•s^−1^) was estimated based on distance (25 m) and T25. A chrono-frequency meter (FINIS 3x300, Finis Inc., USA) was used to assess the stroke rate (SR, Hz) according to three consecutive stroke cycles between the defined section (i.e., 13 m). The stroke length (SL, m) and the stroke index (SI, m^2^•s^−1^) were estimated as SL = v/SR and SI = v • SL, respectively.

### Statistical Analysis

The Shapiro-Wilk test was used to assess the normality of data. A log transformation (log10) was performed if the assumption of normality was violated. Data were back-transformed from the log scale for presentation of the results. The mean and one standard deviation (M ± 1 SD) were computed as descriptive statistics. An independent *t*-test was used to compare boys with girls, and the variation between M_i_ was analyzed with repeated measures ANOVA followed by the Bonferroni *post-hoc*. The assumptions of ANOVA were tested, and Greenhouse-Geisser correction was considered when the assumption of sphericity was violated. Partial Eta Squared (η_p_^2^) was considered an effect size measure and interpreted as reported elsewhere (Ferguson, 2009): no effect if 0 < η_p_^2^ ≤ 0.04; a minimum effect if 0.04 > η_p_^2^ ≤ 0.25; a moderate effect if 0.25 > η_p_^2^ ≤ 0.64; and a strong effect if η_p_^2^ > 0.64. The percentage of variation (∆) between M_i_ was calculated (e.g., [M_1_ – M_2_] / [M_1_] • 100). The associations between performance (v25) and the remained variables at the same M_i_ were also analyzed with the Pearson Correlation Coefficient (r) being interpreted as high if r ≥ 0.60, moderate if 0.30 ≥ r < 0.60 or low if r < 0.30 (Malina, 2001). All statistical analyses were performed using the SPSS software (v.27, IBM, SPSS Inc., Chicago, IL, USA) and GraphPad Prism (v.9, GraphPad Software, San Diego, CA, USA). Statistical significance was set at *p* ≤ 0.05.

## Results

Girls and boys were pooled and analysed together as no differences were found between them (*p* > 0.05). The swimmer’s MO was categorized as pre- and mid-PHV (–1.13 ± 0.74) at M_1_. The effects of a full traditional competitive season in all variables are presented in Table 1. A minimum time effect was found in all variables except for anthropometrics, where the stature, the arm span, HSA D and ND changed throughout the various M_i_ with a moderate-strong effect.

**Table 1:** Effects of the full competitive season on in-water force, performance, kinematics and anthropometric variables of young swimmers

Variables	Time effect			Moments (M ± SD)			
	*F*	*p*	η_p_^2^	M_1_	M_2_	M_3_	M_4_
In-water force							
F_PEAK_ D, N	3.956	0.019	0.14	45.96 ± 10.48	50.82 ± 12.31	48.77 ± 13.88	53.41 ± 17.23
F_PEAK_ ND, N	7.206	<0.001	0.23	45.23 ± 11.28	51.37 ± 13.53	53.97 ± 17.23	55.50 ± 18.85
SF_PEAK_ D, N	3.332	0.024	0.12	46.48 ± 13.49	52.46 ± 14.48	52.91 ± 16.98	55.58 ± 19.11
SF_PEAK_ ND, N	3.946	0.012	0.14	47.74 ± 11.67	55.74 ± 14.51	55.26 ± 17.81	57.51 ± 23.62
SyI, %	3.810	0.014	0.14	17.13 ± 12.42	19.03 ± 14.74	16.38 ± 15.39	28.42 ± 17.92
Performance and kinematics							
T25, s	13.739	<0.001	0.36	17.72 ± 1.71	16.89 ± 1.40	17.04 ± 1.59	16.50 ± 1.50
v25, m•s^−1^	13.489	<0.001	0.36	1.42 ± 0.14	1.49 ± 0.12	1.48 ± 0.14	1.53 ± 0.14
SR, Hz	1.186	0.321	0.05	0.79 ± 0.10	0.81 ± 0.06	0.82 ± 0.08	0.82 ± 0.08
SL, m	0.998	0.399	0.04	1.81 ± 0.21	1.85 ± 0.17	1.82 ± 0.15	1.86 ± 0.12
SI, m^2^•s^−1^	6.665	<0.001	0.22	2.57 ± 0.44	2.78 ± 0.43	2.70 ± 0.40	2.85 ± 0.36
Anthropometrics							
Body mass, kg	2.917	0.087	0.11	48.13 ± 8.63	48.12 ± 7.49	49.11 ± 7.73	49.37 ± 7.55
Stature, cm	58.672	<0.001	0.71	156.59 ± 8.07	157.80 ± 8.05	158.74 ± 8.22	160.17 ± 8.31
BMI, kg•m^−2^	1.340	0.268	0.05	19.57 ± 2.96	19.26 ± 2.25	19.43 ± 2.25	19.18 ± 2.10
Arm span, cm	87.967	<0.001	0.79	156.71 ± 9.84	157.98 ± 9.98	160.50 ± 10.32	162.32 ± 10.62
HSA D, cm^2^	40.801	<0.001	0.63	101.36 ± 12.09	105.72 ± 13.02	109.03 ± 14.31	112.08 ± 16.60
HSA ND, cm^2^	47.210	<0.001	0.66	101.46 ± 13.48	106.39 ± 14.23	109.50 ± 14.94	112.17 ± 16.16

D, dominant; ND, non-dominant; M_i_, moments; F_PEAK_, mean peak force; SF_PEAK_, subsequent peak force; SyI, symmetry index; T25, time of 25m; v25, velocity of 25m; SR, stroke rate; SL, stroke length, SI, stroke index; BMI, body mass index; HSA, hand surface area.

**Figure 2:**
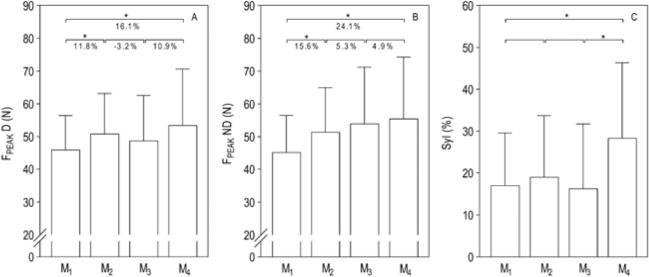
. Effects of the competitive season on swimmers’ in-water force and symmetry. Panel A and B, mean peak forces for dominant (F_PEAK_ D) and non-dominant (F_PEAK_ ND) limbs; Panel C, symmetry index (SyI). * *p* ≤ 0.05; ** *p* ≤ 0.01

**Figure 3:**
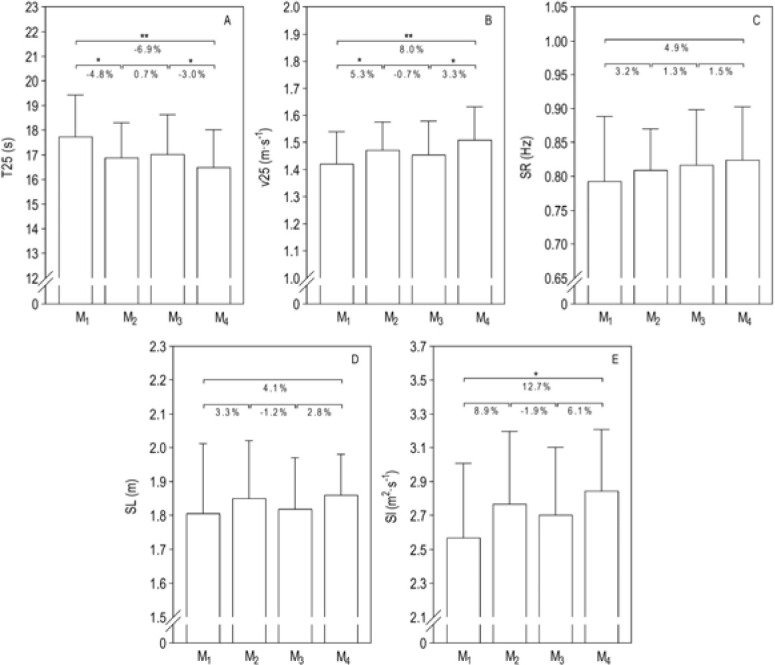
. Effects of the competitive season on swimmers’ performance and kinematics. Panel A, time of 25 m (T25); Panel B, velocity of 25 m (v25); Panel C, stroke rate (SR); Panel D, stroke length (SL); Panel E, stroke index (SI). * *p* ≤ 0.05; ** *p* ≤ 0.01

**Figure 4:**
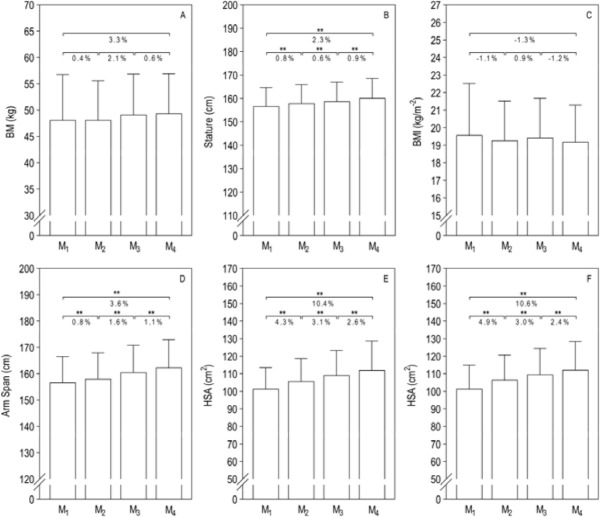
Effects of the competitive season on swimmers’ anthropometrics. BMI, body mass index; D, dominant; ND, non-dominant; HSA, hand surface area. * *p* ≤ 0.05; ** *p* ≤ 0.01

Repeated measures between M_i_ and the variation (∆) are shown in Figures 2–4. There were performance improvements in the first and third macrocycles. Regarding kinematics, while the SI increased from the beginning to the end of the season, both SL and SR remained unchanged over time.

The Pearson Correlation Coefficients between performance and the remained variables are shown in Table 2. Moderate to high associations were found with F_PEAK_ D, F_PEAK_ ND, SI, stature, the arm span, HSA D and HSA ND throughout the competitive season. Performance showed a moderate association with SR in M_1_ and a high association in M_3_ and M_4_. Regarding SL, a high association was found in M_2_. BM showed a moderate association in M_3_ and M_4_.

**Table 2:** Associations between the performance (v25) and the in-water force, kinematics, and anthropometrics variables according to each M_i_

Variables	v25			
	M_1_	M_2_	M_3_	M_4_
F_PEAK_ D, N	0.655**	0.507**	0.492*	0.458*
F_PEAK_ ND, N	0.677**	0.707**	0.705**	0.577**
SyI, %	0.395	−0.115	−0.153	−0.203
SR, Hz	0.540**	0.309	0.606**	0.727**
SL, m	0.316	0.639**	0.376	0.300
SI, m^2^•s^−1^	0.766**	0.895**	0.850**	0.863**
BM, kg	0.291	0.318	0.433*	0.401*
Stature, cm	0.585**	0.537**	0.524**	0.625**
Arm Span, cm	0.541**	0.537**	0.565**	0.642**
HSA D, cm^2^	0.482*	0.667**	0.661**	0.691**
HSA ND, cm^2^	0.508**	0.705**	0.697**	0.736**

v25, swimming velocity; M_i_, moments; F_PEAK_, mean peak force; D, dominant; ND, non-dominant; SyI, symmetry index; SR, stroke rate; SL, stroke length; SI, stroke index; BM, body mass; HSA, hand surface area. * p ≤ 0.05; ** p ≤ 0.01

## Discussion

This study aimed to analyze changes in young swimmers' performance-related variables throughout a competitive season. The main finding was that in-water force and performance improved over the competitive season. Despite the natural anthropometric growth, the stroke index was the single variable that changed between the beginning and the end of the season, translating into greater swimming efficiency. Performance showed association with in-water forces, stroke efficiency or anthropometric features when different time points of the season were considered.

Young swimmers are able to increase in-water forces (16–24% ∆) over a competitive season with increments being more evident in the first macrocycle of the season (~12% ∆). Although training at early ages should focus on technique, it cannot be discarded that a given distance should be covered in the shortest possible time (Morais et al., 2021). Thus, any performance enhancement is underpinned by an increase in the water force production while attempting to diminish drag (Barbosa et al., 2013). Few studies highlighted the long-term effects on swimmers in-water forces (Santos et al., 2021) being mainly related to the effects of using propulsion devices (Barbosa et al., 2020), warm-up routines or detraining (Neufer et al., 1987; Ruiz-Navarro et al., 2022; Szczepan et al., 2022). However, to date, no study has been conducted to understand the force behavior throughout a full competitive season. The deterministic models point out that in-water forces could also be determined by strength levels (Barbosa et al., 2013). At least for young swimmers, swimming training over a competitive season promotes a progressive increase in strength of shoulder rotators (Batalha et al., 2013). Thus, it can be argued that a similar trend seems to occur for in-water forces as it happens in dry-land strength. Although dry-land strength gains seem to have a little transfer to water force production (Amaro et al., 2017), further studies are needed to clarify if, at any moment of the season, this transfer could happen in a strongest way.

Another important finding was related to limb’s dominance, as a higher proportion to increase force over the season occurred in the non-dominant side. In fact, a higher force on the contralateral side can be achieved when swimmers breathe unilaterally (Tourny-Chollet et al., 2009). At least in dry-land, an increase in muscular imbalances in young swimmers has been reported for internal and external shoulder rotators after a full competitive season (Batalha et al., 2013). The same trend seems to occur with the forces, as a higher contralateral asymmetric motion (i.e., higher SyI) was found at the end of the season. Still, the degree of imbalance may dissipate over the detraining period where the SyI may decrease after the summer break (–46.7% ∆) (Santos et al., 2023). Therefore, it can be stated that a competitive season can lead to a more asymmetric force pattern in each stroke cycle. Nevertheless, future research should try to understand the effects of breathing and dry-land strength on in-water forces, establishing a cause-effect relationship in the different stages of the season.

The main performance improvements (8.0% overall over the season) occurred in the first (5.3%) and third (3.3%) macrocycles, while SI changes were only observed between the beginning and the end of the season (12.7%). Previous literature displayed similar improvements in 100 m (Morais et al., 2013), 200 m (Fiori et al., 2022b) and 400 m front crawl (Ferreira et al., 2021; Lätt et al., 2009a, 2009b; Zacca et al., 2020) throughout a competitive season. Increases in velocity can be reached using different individual SR-SL relationships in both adults and young swimmers (Barbosa et al., 2008, 2010). However, improving SR while maintaining SL or improving SL while maintaining SR is a challenge at such early ages (Moreira et al., 2014). The present follow-up study showed that young swimmers tended to improve SR and SL in most stages of the season, but without statistical meaning. Morais et al. (2013) and Lätt et al. (2009a) also noticed no differences in SR for girls and boys between the beginning and the end of the season. However, when maximum technical skill is reached, changes in stroke mechanics are trivial (Costa et al., 2012b). Meanwhile, changes in motor control due to the growth could influence stroke mechanics and efficiency in young swimmers (Seifert et al., 2004) and some kind of “relearning” of stroke mechanics should be considered whenever growth spurts occur.

The second macrocycle (M_2_–M_3_) showed a non-linear change and is worthy of particular awareness. Performance (−0.7%), F_PEAK_ D (−3.2%), SL (−1.2%) and SI (−1.9%) were reduced at this specific time point. Similar impairment in performance (i.e., velocity) was found in the time to complete the front crawl 100 m (Morais et al., 2013). Here, the training periodization could impact performance and technical variables in a given time. Performance was highly associated with SL only in M_2_ (end of the first macrocycle), while in remaining time points it demonstrated a moderate to high association with SR. At some moment during the annual plan, young swimmers might not be completely effective in getting technical adaptations while working for different distances (Strzala and Tyka, 2007). Despite that, some increases in SR, associated with a slight decrease in SL, should not be considered ineffective (Huot-Marchand et al., 2005). However, at such young ages, the technical adjustment in individual SR-SL relationships seems difficult to acquire and to maintain an optimal velocity and efficiency (Strzala and Tyka, 2007). One might consider that swimmers of the present study were under the same phenomenon since the SI showed a non-linear change. However, as the SI was retrieved by estimation from kinematic variables, the changes should occur with the same trend. Still, the reason why young swimmers are more efficient after a training season is probably due to an improvement in technique and changes in anthropometric traits.

After 48 weeks, swimmers were taller (3.6 cm) with a larger arm span (5.6 cm) and a greater hand surface area (D: 10.7 cm^2^; ND: 10.7 cm^2^). Such variables also showed a high association with performance. Some variations during circumpubertal years are essentially linked to growth maturation (Malina et al., 2004). Previous studies conducted with young swimmers in different time periods also reported similar anthropometric changes throughout a macrocycle (Ferreira et al., 2019), a full competitive season (e.g., Fiori et al., 2022a, 2022b; Lätt et al., 2009a, 2009b) and a detraining period (Gryko et al., 2019; Fiori et al., 2022c; Santos et al., 2023).

This study has some limitations which should be acknowledged. The sample size was too small to allow generalization, and therefore the conclusions should be interpreted with caution. The distance used to assess swimming performance was short and the pressure sensors used only allowed to estimate the resultant force instead of the effective propulsive force. On the other hand, swimmers were initially characterized according to biological maturation, but this was assessed using an indirect method. Thus, future studies should attempt to understand long-term changes in applied forces, namely propulsive force, according to the swimmer’s biological maturation (assessed directly) of the same age group. Despite that girls and boys do not differ in all variables, splitting the data according to the swimmers' sex and maturity offset can be beneficial for tracking swimmers' features and to analyze trends. Moreover, the effect of in-water forces and their relationship with performance and dry-land strength in other swimming strokes and distances could also provide a deeper insight into the topic.

## Conclusions

The in-water forces and performance of young swimmers improve over a full competitive season, accompanied by a natural anthropometric growth translating into more efficient swimming. It should be expected that swimming performance would be associated with in-water forces, stroke efficiency or anthropometric features when different moments of the season are considered. This, coaches should be aware that, at a given point of the season, young swimmers may not be able to apply effectively in-water force and shift their technique to desired levels, which may lead to a temporary decrease in efficiency.
